# Fate of Tebuconazole and Trifloxystrobin in Edible Rose Petals: Storage Stability and Human Health Risk Assessment

**DOI:** 10.3390/molecules30193938

**Published:** 2025-10-01

**Authors:** Xiaotong Qin, Jinwei Zhang, Yan Tao, Li Chen, Pingzhong Yu, Junjie Jing, Ercheng Zhao, Yongquan Zheng, Min He

**Affiliations:** 1 Institute of Plant Protection, Beijing Academy of Agriculture and Forestry Sciences, Beijing 100097, China; qinxiaotong0622@163.com (X.Q.);; 2 College of Plant Health and Medicine, Qingdao Agricultural University, Qingdao 266109, China; yqzheng@ippcaas.cn; 3 Beijing Key Laboratory of Environment Friendly Management on Fruit Diseases and Pests in North China, Key Laboratory of Environment Friendly Management on Fruit and Vegetable Pests in North China (Co-Construction by Ministry and Province), Ministry of Agriculture and Rural Affairs, Beijing 100097, China

**Keywords:** edible rose petals, tebuconazole, trifloxystrobin, pesticide residue, risk assessment

## Abstract

This study addresses the absence of maximum residue limits (MRLs) for tebuconazole and trifloxystrobin in edible rose petals in China by systematically evaluating the residue behavior and dietary exposure risks of these fungicides. An analytical method based on QuEChERS sample preparation coupled with UPLC–MS/MS was developed for the simultaneous determination of tebuconazole, trifloxystrobin, and its metabolite CGA321113 in fresh and dried rose petals. Field trials under the highest application conditions (184 g a.i./hm^2^, applied twice) were conducted to investigate residue dissipation dynamics, storage stability, processing concentration effects, and transfer behavior during brewing. Results indicated that the target compounds remained stable in rose petals for 12 months at –20 °C ± 2 °C. The drying process significantly concentrated residues due to the hydrophobic nature of the compounds, with enrichment factors ranging from 3.0 to 3.9. Brewing tests further confirmed low transfer rates of tebuconazole, trifloxystrobin, and CGA321113 into the infusion, consistent with their low water solubility and high log K_ow_ values. Residue dissipation followed first-order kinetics, with half-lives of 1.9–2.9 days for tebuconazole and 1.2–2.7 days for trifloxystrobin. Dietary risk assessment showed an acceptable risk for trifloxystrobin (RQ = 22.7%) but a high risk for tebuconazole (RQ = 175.1%). It is recommended to set the MRL for both tebuconazole and trifloxystrobin in edible roses at 15.0 mg/kg. This standard ensures consumer safety while accommodating agricultural needs and aligns with international regulations. For the high-risk pesticide tebuconazole, measures such as optimizing application strategies and promoting integrated management should be implemented to mitigate residue risks.

## 1. Introduction

For centuries, edible rose petals have been used to make jams, teas, wine, cakes, flavor extracts, and candies [1]. Studies have shown that rose-petal-based products possess various pharmacological activities, including anti-aging and antidepressant effects [2]. Additionally, long-term consumption may promote blood circulation, protect the liver, and regulate the endocrine system [3]. With globalization and increasing consumer awareness of healthier lifestyles, rose petals have gained popularity in the food industry as nutritional supplements [4]. As consumer eating habits evolve, rose petals are emerging as key ingredients in the nutrition and food industries [5].

In response to the growing domestic and international demand for rose-based products, the cultivation area dedicated to edible roses in China continues to expand steadily. Predominant commercial cultivars include Rosa ‘Miaofengshan’ (Beijing), Rosa ‘Pingyin’ (Shandong Province), Rosa ‘Kushui’ (Shanxi Province), and Rosa ‘Mo-red’ (Yunnan Province), all of which have been cultivated for over a millennium [6]. Owing to their distinctive qualities, both the fresh petals and processed derivatives of these roses enjoy considerable market popularity. However, pests and diseases pose significant challenges during their cultivation, among which powdery mildew represents one of the most serious threats. The application of chemical pesticides remains an effective approach to disease management in edible rose production.

A 30% trifloxystrobin–tebuconazole suspension concentrate (containing 20% tebuconazole and 10% trifloxystrobin), developed by Shaanxi Xiannong Biotechnology Co., Ltd. (Weinan, China), is a novel and highly effective agent for controlling powdery mildew in edible roses. Although both tebuconazole and trifloxystrobin are classified as low-toxicity pesticides and demonstrate high efficacy, their extensive use may still pose potential risks to plants, ecosystems, and human health [7,8]. Moreover, CGA321113 (the primary acid metabolite of trifloxystrobin) demonstrates greater aqueous mobility and solubility compared to the parent compound, raising further concerns about its environmental fate and potential health effects [9]. Therefore, it is imperative to evaluate the residual levels of trifloxystrobin (including its metabolite CGA321113) and tebuconazole in edible rose petals to ensure the safety of rose-derived products for human consumption.

In recent years, research on tebuconazole residues in crops has primarily focused on rice [10], wheat [11], cabbage [12], grapes [13], mango [14], and pomegranate [15], while studies on trifloxystrobin residues have involved crops such as cucumber and cowpea [16], tomato [17], chili and apple [18], and Dendrobium officinale [19]. In contrast, research on edible roses has largely concentrated on food processing, nutritional and health benefits, as well as disease and pest control, with systematic studies on pesticide residues remaining relatively limited.

Currently, there is a severe shortage of registered pesticides specifically approved for use on edible roses, and no standardized application protocols have been established. This regulatory gap has resulted in increasingly prominent risks associated with pesticide residues. In the absence of professional guidance, growers frequently apply pesticides intended for ornamental roses to edible varieties, further exacerbating residue contamination. Moreover, as rose petals are often consumed directly or through brewing, residual pesticides may exhibit higher bioavailability and be more readily absorbed by the human body, thereby elevating the potential for long-term cumulative health risks.

Against this backdrop, in-depth research on pesticide residues in edible roses is of both significant importance and urgency. This study systematically investigated the degradation kinetics, terminal residues, storage stability, and transfer behavior during the brewing of tebuconazole and trifloxystrobin in edible roses, along with a comprehensive dietary exposure risk assessment. The key innovation of this work lies in its substantial expansion of the systematic baseline data on pesticide residues in edible rose petals, thereby providing critical scientific support for the establishment of MRLs for these fungicides in edible roses in China. The findings are expected to offer practical guidance for growers in implementing rational pesticide application practices, while also serving as a technical foundation for regulatory authorities in the development of residue standards and risk management strategies.

## 2. Results and Discussion

### 2.1. Method Validation

Tebuconazole, trifloxystrobin, and CGA321113 demonstrated excellent linearity within the concentration range of 0.5–500 ng/mL, with coefficients of determination (R^2^) exceeding 0.99 for all analytes. The absence of significant interfering peaks at the retention times of the target analytes in blank matrix samples confirmed the high selectivity of the method. Matrix effects for tebuconazole, trifloxystrobin, and CGA321113 were effectively controlled within the acceptable range of ≤±20%, enabling successful quantification using a solvent-based calibration curve with the external standard method.

Method accuracy and precision were evaluated through fortification recovery experiments at three concentration levels (10, 100, and 1000 μg/kg). In fresh rose petals, the mean recoveries of tebuconazole, trifloxystrobin, and CGA321113 were 92.5–99.8%, 90.8–107.2%, and 93.8–105.3%, respectively. The corresponding recoveries in dried petals ranged from 94.9–111.0%, 94.0–106.5%, and 97.6–105.9%. Intra-day precision (expressed as RSDₐ, *n* = 5) ranged from 1.6% to 4.4%, while inter-day precision (RSDᵦ, *n* = 15) varied between 1.3% and 8.4%, indicating satisfactory method precision.

In rose tea infusions and their corresponding residues, the recoveries of tebuconazole, trifloxystrobin, and CGA321113 were 88.7–104.2%, 85.3–102.6%, and 90.1–103.9% (tea liquor), and 91.5–106.8%, 89.4–105.1%, and 92.7–107.4% (rose petals), respectively. All relative standard deviations (RSDs, *n* = 5) were below 6.8%. These results are in compliance with internationally accepted residue analysis guidelines, which specify acceptable recovery ranges of 70–120% and RSDs ≤ 15%, confirming the reliability of the method for the quantification of target analytes in rose tea products.

For samples exceeding the linear calibration range, a validated 10- to 20-fold dilution was applied, which consistently yielded satisfactory recoveries. The method exhibited high sensitivity, with limits of detection (LOD) and quantification (LOQ) determined to be 0.003 mg/kg and 0.01 mg/kg, respectively, based on signal-to-noise (S/N) ratios of 3 and 10.

**Figure 1:**
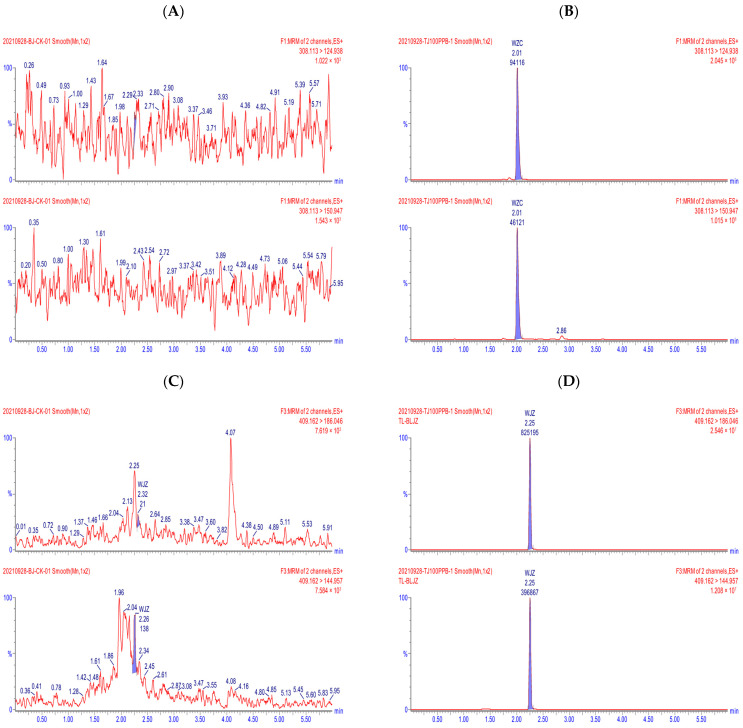

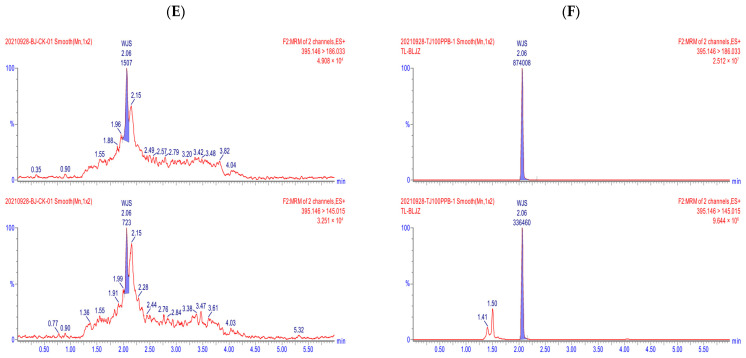
MRM chromatograms of tebuconazole, trifloxystrobin, and CGA321113 in blank (**A**,**C**,**E**) and 0.1 mg/kg-spiked (**B**,**D**,**F**) rose petal samples.

All validated parameters—including selectivity, linearity, accuracy, precision, sensitivity, and dilution integrity—conformed to the acceptance criteria [20], demonstrating that the method is fit for the purpose of quantifying the target analytes in rose petal matrices. Representative multiple reaction monitoring (MRM) chromatograms are provided in Figure 1.

### 2.2. Dissipation Dynamics of the Fungicides

**Figure 2:**
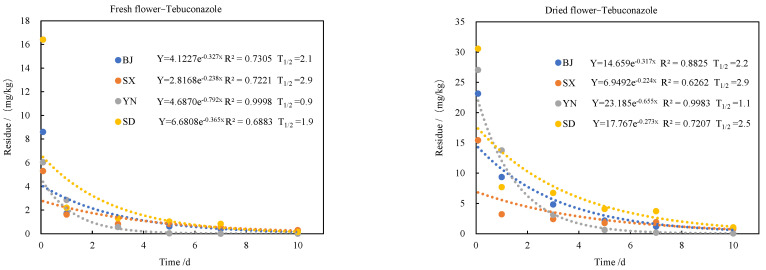

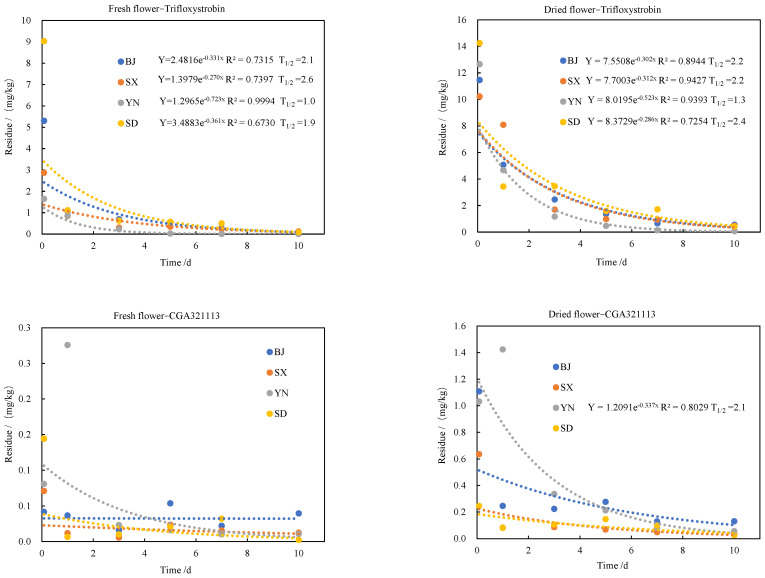
Dissipation kinetics and half-lives of tebuconazole, trifloxystrobin, and metabolite CGA321113 in edible roses from major production regions in China: Beijing (BJ), Shanxi (SX), Yunnan (YN), and Shandong (SD).

The dissipation kinetics of tebuconazole, trifloxystrobin, and its metabolite CGA321113 in edible roses were investigated across four major production regions in China (Beijing, Shanxi, Yunnan, and Shandong), as presented in Figure 2. Initial residue concentrations, measured 2 h after application, exhibited notable regional variation: tebuconazole residues ranged from 5.30 to 16.41 mg/kg, while trifloxystrobin ranged from 1.64 to 8.93 mg/kg.

Significant differences in the initial concentration were observed among the experimental sites, primarily attributable to variations in post-application environmental conditions. Although controllable factors (including application dose, frequency, equipment, and crop growth stage) were strictly standardized across all sites, uncontrollable environmental variables still exerted a considerable influence on the initial deposition amount. For instance, at the Shandong site, sunny conditions and elevated temperatures following application favored effective adhesion and rapid drying of the spray solution on the crop surface. In contrast, rainfall prior to application at the Yunnan site may have resulted in higher surface humidity and reduced stomatal opening, thereby diminishing initial pesticide adhesion and penetration, ultimately leading to a significantly lower initial concentration compared to Shandong. Furthermore, discrepancies in rose cultivars, plant height, planting density, and above-ground biomass among regions may also have contributed to the variability in initial deposition. It is particularly noteworthy that the higher residue levels observed in Shandong could be associated with the relatively shorter plant stature and the BBCH 61 growth stage (full flowering) at the time of sampling. During this stage, rose buds account for a higher proportion of the plant material. Given their larger specific surface area per unit weight, the buds are likely to be more prone to pesticide adsorption. Collectively, these factors underscore the inherent uncertainties associated with environmental and biological variability in field trials.

The metabolite CGA321113 was detected at concentrations between 0.04 and 0.14 mg/kg, accounting for 0.75% to 4.62% of the parent compound (trifloxystrobin) at this initial stage. Although these proportions were relatively low, the rapid emergence of CGA321113 within two hours of application indicates swift initial degradation of trifloxystrobin on the plant surface. Regional differences in the metabolite-to-parent ratio further suggest that local environmental conditions influence the degradation kinetics. Notably, the dissipation of CGA321113 did not conform to first-order kinetics, implying a more complex pattern of formation and degradation compared to the parent compounds. This deviation is likely due to its concurrent generation from trifloxystrobin and simultaneous degradation. Under field conditions, the residue behavior of trifloxystrobin and its metabolite CGA321113 in gherkins differed significantly from that observed in roses. No metabolite CGA321113 was detected in gherkins within 2 h after application [21], which may be attributed to differences in the crop matrix. Furthermore, the initial deposition concentration of trifloxystrobin in roses (5.30–16.41 mg/kg) was significantly higher than that in gherkins (0.83 mg/kg). This concentration discrepancy may further influence the metabolic rate and detectable residue levels.

In contrast, the dissipation of both tebuconazole and trifloxystrobin followed first-order kinetics, with half-lives of 1.9–2.9 days and 1.2–2.7 days, respectively. Approximately 90% of both parent compounds dissipated within 7 days, indicating relatively rapid degradation under field conditions. The significance of differences in the degradation kinetic parameters (including the degradation rate constant *k* and t_1/2_) of tebuconazole and trifloxystrobin among different regions was analyzed using a one-way analysis of variance (ANOVA) followed by Duncan’s new multiple range test. The results indicated that no significant differences were observed in either the degradation rate constants (k) or half-lives (t_1_/_2_) among the four regions (*p* > 0.05).

Comparative analysis with the existing literature reveals substantial differences in the half-lives of tebuconazole across crops. The shortest half-lives were reported in cucumbers (1.1–3.2 days) [21,22], tomatoes (1.2–1.7 days) [23], and cabbage (2.7–2.8 days) [12]; moderate half-lives were observed in strawberries (6.3 days) [24], paddy plants (5.7–13.9 days) [25], green onions (9.4–9.9 days) [26], bananas and apples (7.3–8.7 days) [27,28], and pears (8.8–15.4 days) [29,30]; and the longest half-life was recorded in jujube (33.0 days) [31]. Similarly, trifloxystrobin half-lives varied by crop. Shorter half-lives occurred in tomatoes (1.08–2.77 days) [17,18,23] and cucumbers (2.4–3.1 days) [21,22], while a longer t_1/2_ was found in onions (4.7–4.8 days) [32], and rice and wheat plants (5.8–7.6 days) [10,11]. This comparative assessment demonstrates that the dissipation rates of both fungicides in rose matrices are significantly faster than those in most fruits and cereal crops, but show no statistically significant difference compared to their dissipation in tomatoes and cucumbers.

### 2.3. Terminal Residues of the Fungicides

In a dietary risk assessment for plant-derived foods, the residue definition for trifloxystrobin includes the sum of trifloxystrobin and its metabolite CGA321113, expressed as the parent compound. Accordingly, all subsequent references to “trifloxystrobin concentration” in this part refer to the combined concentrations of trifloxystrobin and CGA321113.

Terminal residue trials showed that concentrations of tebuconazole ranged from 0.01–1.05 mg/kg in fresh rose petals to 0.03–4.09 mg/kg in dried petals, while residues of trifloxystrobin ranged from 0.01–0.61 mg/kg in fresh petals to 0.12–1.96 mg/kg in dried petals. The drying process led to an average enrichment factor between 3.0 and 3.9. This concentration effect is consistent with previously reported behavior during the drying of medicinal and edible crops such as honeysuckle [33], tea [34,35], and rose [36]. Mechanistic analysis indicates that both tebuconazole and trifloxystrobin are highly hydrophobic, with aqueous solubilities of 36 mg/L and 0.61 mg/L, respectively (20 °C, pH 5–9), which limits their mobility in the aqueous phase during drying. Concurrent water evaporation and volumetric reduction in the plant matrix result in significant physical concentration of pesticide residues, leading to markedly elevated residue levels in dried rose petals.

**Figure 3:**
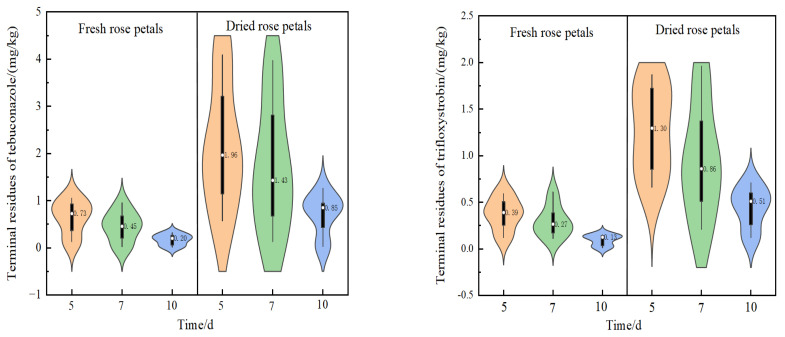
Terminal residue of tebuconazole and trifloxystrobin in fresh and dried rose petals at different PHIs.

Figure 3 illustrates the residue concentrations of tebuconazole and trifloxystrobin across different PHIs. At each PHI (5, 7, and 10 days), residue levels of both fungicides were significantly higher in dried petals than in fresh material, confirming the concentrating effect of dehydration. For instance, at the 5-day PHI, the STMR of tebuconazole increased from 0.73 mg/kg in fresh petals to 1.97 mg/kg in dried petals—a increase of approximately 2.7 fold. A decreasing trend in STMR values over time was observed for both compounds in both matrices. In fresh petals, the STMR of tebuconazole declined from 0.73 mg/kg at 5 days to 0.46 mg/kg at 7 days, and further to 0.20 mg/kg at 10 days. A similar dissipation pattern was noted for trifloxystrobin. Notably, the STMR of tebuconazole in dried petals at the 10-day PHI (0.85 mg/kg) still exceeded that in fresh petals at the 5-day PHI (0.73 mg/kg), clearly indicating that drying delays residue dissipation.

The highest residue (HR) is a critical parameter in acute dietary exposure assessment. The maximum residue level of tebuconazole observed in this study was 4.09 mg/kg in dried petals at the 5-day PHI, highlighting the importance of accounting for processing-induced concentration when establishing MRLs and conducting dietary risk assessments for dried products.

Based on the terminal residue data, it is recommended that application of 30% tebuconazole–trifloxystrobin SC to edible roses be avoided during the flowering stage to minimize residue accumulation. If disease control measures are required, a PHI of no less than 7 days must be observed. Furthermore, future risk assessments should explicitly consider the concentrated residue levels in dried rose products to ensure consumer safety.

### 2.4. Assessment of the Storage Stability of the Fungicides

The experiment used samples spiked at a concentration of 1.0 mg/kg, stored under cold conditions at −20 ± 2 °C for 12 months. The storage stability results for tebuconazole, trifloxystrobin, and CGA321113 are presented in Table 1.

In fresh rose petals, the measured concentrations of tebuconazole, trifloxystrobin, and CGA321113 ranged from 0.87 ± 0.05 to 1.12 ± 0.07 mg/kg, 0.91 ± 0.05 to 1.05 ± 0.06 mg/kg, and 0.87 ± 0.05 to 1.12 ± 0.06 mg/kg, respectively. Similarly, in dried petals, the concentrations varied between 0.94 ± 0.05 and 1.09 ± 0.06 mg/kg for tebuconazole, 0.95 ± 0.05 and 1.09 ± 0.06 mg/kg for trifloxystrobin, and 0.97 ± 0.05 and 1.11 ± 0.06 mg/kg for CGA321113. The degradation rates (DRs) for tebuconazole ranged from −11.2% to 7.7% in fresh petals and from −8.9% to 6.2% in dried petals. For trifloxystrobin, the DR varied between −8.3% and 8.7% (fresh) and between −8.1% and 8.1% (dried). CGA321113 exhibited slightly broader variation, with DR values ranging from −12.1% to 13.0% in fresh petals and from −10.9% to 3.4% in dried petals. Importantly, all observed degradation rates remained well below the 30% stability threshold, indicating no significant compound deterioration over time.

QC recoveries consistently fell within the acceptable range of 84.7–108.9%, thereby confirming the reliability and accuracy of the analytical method throughout the stability study. These results collectively demonstrate that tebuconazole, trifloxystrobin, and CGA321113 maintain good stability in both fresh and dried rose petals for up to 12 months under the applied storage conditions.

### 2.5. Effect of Brewing on the Fungicide Residues

The experimental samples comprised fresh petals collected 2 h after pesticide application and dried petals processed under a standardized drying protocol. All samples were cryogenically homogenized into a fine powder using dry ice. Initial residue analysis showed that the concentrations of tebuconazole, trifloxystrobin, and CGA321113 in fresh petals were 16.41 mg/kg, 9.03 mg/kg, and 0.14 mg/kg, respectively. In dried petals, the residue levels increased to 28.94 mg/kg, 13.77 mg/kg, and 0.25 mg/kg. None of the target compounds were detected in the tea infusions brewed from either fresh or dried petals over brewing times of 1, 2, 5, 10, and 30 min (method detection limit < 0.01 mg/L). The concentrations of tebuconazole, trifloxystrobin, and CGA321113 in the petals after brewing are summarized in Table 2. The residue levels of all three compounds gradually decreased with extended brewing time, which may be attributed to thermal degradation or shifts in the continuous dissolution–adsorption equilibrium under high-temperature conditions.

Tebuconazole and trifloxystrobin possess low water solubility (36 mg/L and 0.61 mg/L, respectively) and high octanol–water partition coefficients (log Kow 3.7 and 4.5, respectively), properties that promote their retention in the waxy cuticle and lipid-rich tissues of rose petals rather than transfer into the aqueous infusion. To assess the generalizability of this behavior, rose samples from three additional regions were brewed for 30 min. Consistent with the initial results, no target compounds were detected in the infusions, under the applied high-temperature (90 °C) and low liquid-to-solid ratio (10:1) conditions. Although Yanli Bian et al. [36] also reported that pesticides with low water solubility and high Kow generally exhibit low transfer rates, they detected tebuconazole and trifloxystrobin in infusions (data for metabolite CGA321113 were not provided). This discrepancy may stem from differences in sample preparation: Bian et al. [36] used whole dried rose buds, whereas the present study employed powdered material, whose greater specific surface area may enhance the adsorption of hydrophobic pesticides, thereby further limiting their release into the infusion.

### 2.6. Dietary Risk Assessment

In China, both tebuconazole and trifloxystrobin are registered and approved for use on a variety of crops. MRLs for these two fungicides have been established by major regulatory jurisdictions, including the Codex Alimentarius Commission (CAC), China, the United States (USA), Australia, the European Union (EU), Korea, and Japan, for relevant crops. Detailed MRL data are provided in Table 3.

The dietary intake risks of tebuconazole and trifloxystrobin were assessed based on the risk maximization principle. The ADI values, as specified in GB 2763 [37,38], were set at 0.03 mg/kg bw for tebuconazole and 0.04 mg/kg bw for trifloxystrobin. The national estimated daily intake (NEDI) was calculated by integrating authorized application data for the registered crops with the general Chinese average dietary consumption structure. During the assessment, MRLs stipulated in GB 2763 were adopted as the reference standards for corresponding food categories, with the highest MRL applied for items within the same group. For crops without established domestic MRLs, the highest international MRL standards were applied. The resulting NEDI values are summarized in Table 4.

The NEDI values for the general population were 3.3091 mg for tebuconazole and 0.5728 mg for trifloxystrobin, corresponding to risk quotient (RQ) values of 175.1% and 22.7%, respectively. Re-evaluation using the supervised trial median residue (STMR) values from supervised trials on dried rose petals with a 7-day application interval (1.43 mg/kg for tebuconazole and 0.86 mg/kg for trifloxystrobin) resulted in reduced RQ values of 166.5% and 16.0%, respectively. When STMR values for fresh rose petals (0.46 mg/kg for tebuconazole and 0.27 mg/kg for trifloxystrobin) were applied, the RQs further decreased to 165.9% and 15.7%. Since edible roses are classified under “salt” in the Chinese food classification system and the daily intake of salt by the general population is very low, setting the MRLs for both fungicides in edible roses at 15 mg/kg would not significantly affect the overall RQ values. Notably, China has not yet established MRLs for tebuconazole and trifloxystrobin in edible roses, whereas the EU has set relevant standards. Based on the experimental and risk assessment data presented in this study, it is recommended to establish the maximum residue limits (MRLs) for both tebuconazole and trifloxystrobin in edible roses at 15.0 mg/kg.

The assessment results demonstrate that the choice of parameters (either MRLs or the STMR) significantly influences the outcomes of the risk evaluation. Under the most stringent assessment scenario, the dietary exposure risk associated with trifloxystrobin remains substantially below the safety threshold. This indicates that the existing MRLs of trifloxystrobin provide an adequate safety margin, even considering potential future expansions in registered crop uses. Tebuconazole poses a relatively high dietary intake risk, indicating a potential threat to public health. As is shown in Table 4, China’s MRLs for tebuconazole in celery and cabbage are significantly higher than those set by other major regulatory authorities. Analysis shows that if the MRLs for dark-colored vegetables and light-colored vegetables are limited to 5.0 mg/kg and 3.0 mg/kg, respectively, the RQ value can be reduced to 78.6%. This suggests that the current standards for celery and cabbage are perhaps overly lenient. The findings highlight the widespread and extensive use of tebuconazole in China, with its dietary intake risk already at a relatively high level. The findings further emphasize that to effectively mitigate food safety concerns associated with tebuconazole residues, it is imperative to develop and promote technologies for pesticide reduction and harm control, thereby achieving synergistic management of agricultural productivity and food safety.

## 3. Materials and Methods

### 3.1. Chemicals and Reagents

Tebuconazole (CAS Number 107534-96-3, purity 98.1%), trifloxystrobin (CAS Number 141517-21-7, purity 99.3%), and CGA321113 (CAS Number 252913-85-2, purity 99.48%) were purchased from Beijing Yan Hua Yong Le Biotech Company (Beijing, China). A 30% suspension concentrate (SC) formulation containing 20% tebuconazole and 10% trifloxystrobin was obtained from Shanxi Xian Nong Biological Science and Technology Company (Xi’an, China). Graphitized carbon black (GCB, 40–60 µm), primary secondary amines (PSAs, 40–60 µm), and octadecylsilane (C_18_, 40–60 µm) were purchased from Agela Technologies (Tianjin, China). HPLC-grade acetonitrile, methanol, and formic acid (88% purity) were obtained from Dikma Science and Technology Company (Beijing, China). Analytical-grade acetonitrile, sodium chloride (NaCl), and anhydrous magnesium sulfate (MgSO_4_) were purchased from Beijing Chemical Reagent Company (Beijing, China). Ultra-pure water was produced using a Milli-Q system (Bedford, MA, USA).

### 3.2. Field Trials and Drying Process

To investigate the residue dynamics of tebuconazole and trifloxystrobin in edible rose petals under varying climatic conditions, supervised field trials were conducted in four distinct agricultural regions of China (Beijing Municipality, Shanxi Province, Shandong Province, and Yunnan Province) in accordance with *the Guideline for Pesticide Residue Trials in Crops* [20]. Environmental parameters collected from each experimental site throughout the field trials are summarized in Table 5.

A completely randomized block design was implemented, with one treated plot and one blank control plot established at each location. Each plot covered a minimum area of 300 m^2^ to ensure spatial representativeness of the samples. Under field conditions, 30% SC of trifloxystrobin–tebuconazole was applied twice at the recommended maximum dosage (184 g a.i./hm^2^) with a 10-day interval between applications. Pre-harvest intervals (PHIs) of 5, 7, and 10 days were established for final residue evaluation. For dissipation kinetics analysis, fresh petal samples were collected at 2 h, 1 d, 3 d, 5 d, 7 d, and 10 d after the final application.

Owing to the rapid flowering progression and low per-plant biomass characteristics of roses, all sampling procedures were rigorously performed in compliance with the BBCH scale, exclusively within growth stages 61–67 (encompassing late bud development to initial petal abscission). A multi-point random sampling strategy was adopted, wherein healthy, undamaged complete inflorescences (including both buds and unfolded petals) were collected from no fewer than 12 plants per plot, covering various canopy positions (upper, middle, and lower) and orientations (inner and outer). Both dissipation and final residue studies involved the collection of fresh flower samples.

In parallel, a standardized drying procedure was applied to fresh rose petals to simulate representative industrial practices in China’s rose processing industry. The drying process was conducted using a DHG-9075A electric thermostatic drying oven (Shanghai Yiheng Technology Co., Ltd., Shanghai, China) with a programmed stepwise temperature increase: 36 ± 1 °C for 2 h, followed by 40 ± 1 °C for 6 h, then 50 ± 1 °C for 6 h, and finally 60 ± 1 °C for 6 h. This graded thermal protocol was designed to replicate commercial drying conditions while effectively removing moisture and minimizing thermal degradation of pesticide residues. The initial moisture content of the fresh rose petals, calculated based on the mass change before and after drying, was determined to be 70–85% (on a wet weight basis), which is consistent with the typical moisture range observed for fresh rose petals.

For each sampling time point, four independent biological replicates (each ≥1.0 kg) were collected from the treated plot. Blank control samples were obtained during the first and final sampling events. Prior to residue analysis, all samples (both fresh and dried) were homogenized using dry ice to ensure uniformity. To preserve sample integrity, all materials were stored at −20 ± 2 °C until analysis. This systematic approach ensured robust data generation for evaluating residue persistence under different environmental conditions and processing methods.

### 3.3. Storage Stability Test

The reliability of pesticide residue analysis is of critical importance. To ensure analytical accuracy, this study evaluated the storage stability of tebuconazole, trifloxystrobin, and CGA321113 in both fresh and dried rose petals, in accordance with *the Guideline for the Stability Testing of Pesticide Residues in Stored Commodities of Plant Origin* [45].

For the stability assessment, fresh and dried rose petals were fortified with tebuconazole, trifloxystrobin, and CGA321113 at a concentration of 1.0 mg/kg. Two parallel quality control (QC) samples were prepared to monitor method performance. All samples were stored in 50 mL Teflon centrifuge tubes at −20 ± 2 °C to simulate typical storage conditions. To evaluate residue degradation over time, samples were analyzed at predetermined intervals (0, 1, 2, 3, 6, and 12 months) using a randomized sampling approach. Blank samples (including matrix and solvent blanks) were analyzed to confirm the absence of target residues, with response values not exceeding 30% of the spiked samples at the limit of quantification (LOQ, 0.01 mg/kg) in accordance with *the Guideline for Pesticide Residue Trials in Crops.* This precaution effectively eliminated potential interference from inherent matrix components, ensuring that the observed degradation profiles solely reflected the target analytes.

### 3.4. Brewing Process

Fresh and dry edible rose petals treated with 30% trifloxystrobin–tebuconazole SC were used for the brewing test of flower tea. Weighed fresh petals (5.0 g) or dry petals (2.0 g) were placed separately into 150 mL stoppered flasks. Then, 100 mL of boiled purified water was added to each flask, and the caps were immediately sealed. The flasks were shaken at 150 rpm in a 90 °C constant-temperature water bath oscillator to simulate brewing for 1, 2, 5, 15, and 30 min, respectively, with three replicates per time point. Upon reaching the designated time, the flasks were immediately transferred to a cold-water bath and cooled to room temperature. The mixtures were promptly filtered, and the filtrates (tea liquor) were collected and stored in 100 mL glass-stoppered graduated cylinders. The remaining rose petals were blotted dry to remove surface moisture and retained together with the filtrates. All samples were analyzed within 24 h.

### 3.5. Analytical Method

#### 3.5.1. Extraction and Purification

Accurately weighed aliquots of homogenized fresh rose petals (5.0 g), dried rose petals (2.0 g), rose tea infusion (10.0 g) or rose tea infusion leaves (1.0 g) were individually transferred into 50 mL sealed polypropylene centrifuge tubes. Subsequently, 5 mL of ultra-pure water was introduced into tubes containing fresh or dried petal matrices, followed by homogenization via vortex mixing (1.0 min) to achieve complete hydration. All samples were subsequently subjected to solvent extraction using 20 mL of an acetonitrile/formic acid mixture (100:2, *v*/*v*) under rigorous mechanical agitation at 250 rpm for 8.0 min in a temperature-controlled orbital shaker (25 ± 1 °C). Thereafter, 3.0 g of NaCl was added, and the mixture was vortexed for another 2.0 min. The samples were then centrifuged at 4000 rpm for 5.0 min. After centrifugation, 1.5 mL of the supernatant was transferred into a 2.0 mL cleanup tube containing 25 mg of GCB, 25 mg of PSAs, 25 mg of C_18_, and 150 mg of anhydrous MgSO_4_. The purified extract was filtered through a 0.22 µm nylon membrane into a 2.0 mL autosampler vial. For highly concentrated fresh samples, a 20-fold dilution was performed by mixing 0.05 mL of the sample extract with 0.95 mL of acetonitrile. Similarly, for concentrated dried samples, a 10-fold dilution was carried out by adding 0.1 mL of the extract to 0.9 mL of acetonitrile. All prepared samples were analyzed by UHPLC–MS/MS.

#### 3.5.2. UHPLC–MS/MS Analysis

The analysis was conducted using a Waters ACQUITY H-Class ultra-high-performance liquid chromatography system coupled with a Xevo triple–quadrupole tandem mass spectrometer (UPLC-MS/MS, Waters Corp.). A Waters ACQUITY BEH C_18_ analytical column [2.1 mm (diameter) × 100 mm (length) × 1.7 μm (particle size), Waters Corp.] was used, with an injection volume of 1.0 μL. The sample manager temperature was maintained at 15 °C. The mobile-phase flow rate was set to 0.3 mL/min and consisted of solvent A (acetonitrile) and solvent B (0.1% (*v*/*v*) formic acid in water). The column oven was kept at 35 °C to maintain optimal viscosity of the mobile phase. The gradient elution program was as follows: 0–1.0 min, solvent A increased from 45% to 55%; 1.0–3.0 min, solvent A increased from 55% to 80%; 3.0–5.0 min, solvent A remained at 80%; and 5.0–6.0 min, solvent A decreased from 80% to 55%.

Mass spectrometric analysis was conducted using an electrospray ionization (ESI) source operating in positive switching mode. The key ion source parameters were optimized as follows: capillary voltage 3.63 kV, source temperature 150 °C, and desolvation temperature 400 °C. High-purity nitrogen was employed as both a desolvation gas (flow rate: 700 L/h) and cone gas (flow rate: 150 L/h), while high-purity argon was used as a collision gas (flow rate: 0.20 mL/min) in the collision cell. The analytical method utilized the multiple reaction monitoring mode (MRM) with two characteristic ion transitions selected for each target compound: a quantitative transition (precursor ion→product quantifier ion) for calibration curve establishment and concentration determination, and a qualitative transition (precursor ion→product qualifier ion) for compound confirmation. Specifically, tebuconazole (retention time: 2.01 min) was quantified using transition 308.113→124.938 and confirmed by 308.113→150.947; trifloxystrobin (retention time: 2.25 min) was quantified via transition 409.162→186.046 and confirmed by 409.162→144.957; the metabolite CGA321113 (retention time: 2.06 min) was quantified using transition 395.146→186.033 and confirmed by 395.146→145.015. For all actual samples, the relative deviation of ion abundance ratios between target compounds and standard solutions remained within 30%, and retention time shifts were controlled within ±0.1 min.

### 3.6. Data Processing Methods

The degradation rate (DR) of the stored samples was determined using Equation (1):

*DR* = (*C*_0_ − *C_t_*)/*C*_0_ × 100% (1)

where *DR* represents the degradation rate (%), *C_t_* (mg/kg) is the fungicide concentration *t* (days), and *C*_0_ (mg/kg) denotes the initial spiked concentration (*t* = 0).

In the dietary risk assessment of plant-derived foods, the residue of trifloxystrobin is defined as the sum of the concentrations of trifloxystrobin and CGA321113. The calculation is performed using Equation (2):

*C_t_* = *C*_1_ + (M_1/_M_2_*)* × *C*_2_ (2)

where *C*_1_ and *C*_2_ (mg/kg) represent the concentrations of trifloxystrobin and its metabolite CGA321113, respectively, and M_1_ (408.37) and M_2_ (394.34) denote their corresponding molecular weights.

The degradation kinetics of tebuconazole and trifloxystrobin on fresh edible roses were modeled using first-order kinetic equations, and their half-lives were calculated using Equations (3) and (4):

*C_t_* = *C*_0_
*e*^−*kt*^ (3)

*t*_1/2_ = ln (2)/*k* (4)

where *C_t_* (mg/kg) represents the fungicide concentration at time t, *C*_0_ (mg/kg) denotes the initial concentration immediately after application (*t* = 0), *t* (days) is the time after application, *k* is the first-order rate constant, and *t*_1/2_ (days) is the half-life required for the fungicide concentration to reduce to half of its initial value.

To assess the chronic dietary risk and ensure the safe use of tebuconazole and trifloxystrobin on edible rose petals, the risk quotient (*RQ_c_*) was calculated based on the residue concentrations detected in fresh and dried petals. The calculation is performed using Equation (5):

*RQ_c_* = ∑STMR_i_ × F_i_/(ADI × bw) × 100% (5)

where the STMR is the median residue value (mg/kg) from supervised trials, and the ADI (acceptable daily intake) is the toxicological reference value (mg/kg bw), F_i_ represents the daily food consumption (kg/d) based on the Chinese dietary survey, and bw is the average body weight of an adult (63 kg). An *RQ_c_* value of less than 100% indicates that the chronic dietary risk is acceptable. Conversely, an *RQ_c_* value exceeding 100% suggests a potential health risk with unacceptable adverse effects on human health.

## 4. Conclusions

This study developed a multi-residue analytical method for the simultaneous determination of tebuconazole, trifloxystrobin, and its metabolite CGA321113 in fresh and dried rose petals, based on QuEChERS sample preparation coupled with UPLC–MS/MS. In field trials, two applications were made at the recommended dose of 184 g a.i./hm^2^ during the peak incidence period of *powdery mildew*, with a 10-day interval between applications. Samples collected 5–10 days after treatment showed residue concentrations of tebuconazole ranging from 0.01–1.05 mg/kg in fresh rose petals and 0.03–4.09 mg/kg in dried rose petals, while trifloxystrobin residues ranged from 0.01–0.61 mg/kg in fresh rose petals and 0.12–1.96 mg/kg in dried rose petals. Due to their strong hydrophobicity, both fungicides are unlikely to migrate with water during drying. Storage stability tests indicated that tebuconazole, trifloxystrobin, and CGA321113 remained stable after 12 months of storage at –20 ± 2 °C. Meanwhile, water evaporation and matrix shrinkage led to significant concentration of residues, with an average enrichment factor of 3.0–3.9 times in dried rose petals. The dissipation dynamics followed first-order kinetics with half-lives of 1.9–2.9 days for tebuconazole and 1.2–2.7 days for trifloxystrobin. Dietary intake risk assessment revealed that the RQ for trifloxystrobin was 22.7%, which is well below the safety threshold, indicating that its residues do not pose an unacceptable risk to consumers. In contrast, the RQ for tebuconazole reached 175.1%, suggesting a high dietary exposure risk under current conditions. It is recommended to reduce the application frequency in vegetables and fruits, and promote integrated pest management techniques to lower residue concentrations. Based on the residue behavior and risk assessment results, it is proposed to set the MRLs for both tebuconazole and trifloxystrobin in edible roses at 15.0 mg/kg. This value meets agricultural production needs, aligns with international trade standards, and is scientifically justified and feasible.

## Figures and Tables

**Table 1:** Stability of the three compounds in fresh and dried rose petals under cold storage conditions.

Compound	Storage Time	Fresh Rose Petals			Dried Rose Petals		
	(Months)	Concentration (mg/kg)	DR (%)	QC Recovery (%)	Concentration (mg/kg)	DR (%)	QC Recovery (%)
Tebuconazole	0	1.00 ± 0.06	0.5	101.8	0.99 ± 0.05	1.4	89.7
	1	1.12 ± 0.07	−11.2	107.0	0.99 ± 0.05	1.6	100.4
	2	0.92 ± 0.05	7.7	88.6	1.01 ± 0.05	−0.9	99.8
	3	1.01 ± 0.06	−1.4	87.8	0.97 ± 0.05	3.4	93.0
	6	1.00 ± 0.06	0.7	100.6	0.94 ± 0.05	6.2	99.6
	12	1.04 ± 0.06	−9.2	103.5	1.09 ± 0.06	−8.9	108.2
Trifloxystrobin	0	1.05 ± 0.06	−4.3	103.9	1.03 ± 0.05	−2.5	98.4
	1	0.96 ± 0.05	3.8	96.3	0.95 ± 0.05	8.1	96.0
	2	0.94 ± 0.05	6.2	102.7	0.99 ± 0.05	1.4	99.8
	3	0.94 ± 0.05	6.6	102.0	1.05 ± 0.06	−8.1	106.7
	6	1.05 ± 0.06	−4.6	103.9	1.00 ± 0.05	0.4	107.6
	12	0.91 ± 0.05	8.7	98.3	1.09 ± 0.06	−8.3	104.2
CGA321113	0	1.06 ± 0.06	−5.8	101.2	1.07 ± 0.06	−6.9	94.0
	1	1.12 ± 0.06	−12.1	105.0	0.98 ± 0.05	2.0	90.5
	2	1.02 ± 0.05	−1.4	91.4	1.00 ± 0.05	0.7	100.5
	3	1.08 ± 0.06	−7.6	107.9	0.97 ± 0.05	2.9	99.9
	6	1.07 ± 0.06	−6.6	108.7	1.00 ± 0.05	0.6	104.0
	12	0.87 ± 0.05	13.0	108.9	1.11 ± 0.06	−10.9	84.7

**Table 2:** Concentrations of tebuconazole, trifloxystrobin, and CGA321113 in rose petals after brewing.

Brewing Time (min)	Concentration of Rose Petals (mg/kg)					
	Tebuconazole		Trifloxystrobin		CGA321113	
	Fresh	Dried	Fresh	Dried	Fresh	Dried
1	13.10 ± 0.78	23.10 ± 1.39	7.35 ± 0.44	11.20 ± 0.67	0.20 ± 0.012	0.34 ± 0.020
2	12.50 ± 0.75	21.50 ± 1.29	7.10 ± 0.43	10.50 ± 0.63	0.19 ± 0.011	0.32 ± 0.019
5	11.80 ± 0.71	19.20 ± 1.15	6.60 ± 0.40	9.30 ± 0.56	0.17 ± 0.010	0.30 ± 0.018
10	11.00 ± 0.66	16.80 ± 1.01	6.10 ± 0.37	8.20 ± 0.49	0.15 ± 0.009	0.28 ± 0.017
30	10.20 ± 0.61	13.90 ± 0.83	5.75 ± 0.35	6.80 ± 0.41	0.14 ± 0.008	0.27 ± 0.016

**Table 3:** MRL regulatory values for tebuconazole and trifloxystrobin in major trading countries.

Compound	Crop	MRLs (mg/kg)						
		China [37,38]	CAC [39]	USA [40]	Australia [41]	Korea [42]	EU [43]	Japan [44]
Tebuconazole	Sanchi	15.00	/	/	/	/	/	/
	Celery	15.00	5.00	5.00	5.00	5.00	0.50	5.00
	Chinese cabbage	7.00	/	/	/	2.00	0.02	3.00
	Banana	3.00	1.50	0.05	0.20	0.05	1.50	2.00
	Strawberry	2.00	/	/	2.00	0.50	15.00	2.00
	Tomato	2.00	0.70	1.30	0.50	1.00	0.90	1.00
	Citrus	2.00	/	1.00	0.20	3.00	5.00	5.00
	Bitter melon	2.00	0.15	0.40	0.50	0.20	0.15	0.20
	Pepper	2.00	/	1.30	0.50	3.00	0.05	1.00
	Cotton	2.00	2.00	2.00	1.00	/	2.00	2.00
	Apple	2.00	1.00	1.00	0.01	1.00	0.30	1.00
	Grape	2.00	6.00	6.00	5.00	5.00	0.50	10.00
	Peach	2.00	2.00	1.00	0.01	1.00	0.60	2.00
	Sunflower	2.00	0.10	0.10	0.10	/	0.02	0.10
	Cucumber	1.00	0.20	0.40	0.50	0.20	0.60	0.20
	Radish	1.00	/	0.70	0.50	0.30	0.02	0.10
	Welsh onion	0.50	0.15	1.30	2.00	3.00	2.00	0.20
	Pear	0.50	1.00	1.00	0.01	1.00	0.30	5.00
	Rice	0.50	1.50	/	0.20	0.20	1.50	0.05
	Ginseng	0.40	0.40	/	/	0.50	/	/
	Loquat	0.20	/	1.00	0.01	/	0.50	/
	Garlic	0.10	0.10	0.20	0.20	0.10	0.10	0.20
	Peanut	0.10	0.15	/	0.10	0.05	0.15	0.05
	Watermelon	0.10	/	1.00	/	/	0.15	0.20
	Onion	0.10	/	0.20	/	/	0.15	0.20
	Pecan	0.05	0.05	/	0.05	0.05	0.05	0.05
	Sorghum	0.05	/	/	/	7.00	/	/
	Mango	0.05	0.05	1.00	0.01	0.70	0.10	0.10
	Wheat	0.05	0.15	3.00	0.20	0.05	0.30	2.00
	Winter jujube	/	/	/	/	5.00	/	/
	Goji berry	/	/	/	/	/	0.50	/
	Ginger	/	/	/	/	/	/	0.20
	Cowpea	/	/	0.10	0.50	0.50	0.20	3.00
	Honeysuckle	/	/	/	/	/	/	/
	Potato	0.10	/	/	/	0.05	0.02	0.10
	Dwarf lilyturf	/	/	/	/	/	/	/
	Pomegranate	/	/	/	/	/	0.02	/
	Edible rose	/	/	/	/	/	15.00	/
	Dendrobium officinale	/	/	/	/	/	/	/
	Tobacco	/	/	/	/	0.05	/	/
	Red bayberry	/	/	1.00	/	/	/	/
	Maize	/	0.60	0.50	0.70	0.50	0.60	0.60
	Corydalis	/	/	/	/	/	/	/
Trifloxystrobin	Grape	3.00	3.00	2.00	0.50	3.00	0.50	5.00
	Strawberry	1.00	1.00	2.00	2.00	0.70	0.05	1.00
	Tomato	0.70	0.70	0.50	0.70	2.00	0.70	0.70
	Apple	0.70	3.00	5.00	0.70	/	0.70	3.00
	Eggplant	0.70	0.70	0.50	/	0.70	0.70	0.70
	Mandarin orange	0.50	0.50	0.60	2.00	0.50	0.50	3.00
	Cucumber	0.30	/	0.50	0.10	0.50	3.00	0.70
	Watermelon	0.20	/	2.00	/	0.50	0.30	0.30
	Wheat	0.20	0.20	0.05	/	0.15	0.30	0.20
	Banana	0.10	0.05	0.10	0.50	0.05	0.70	0.50
	Onion	0.05	/	0.04	/	0.05	0.01	0.70
	Maize	0.02	/	0.05	/	0.02	0.02	0.05
	Garlic	/	/	0.04	/	0.50	0.01	0.05
	Daylily	/	/	/	/	/	0.01	/
	Cowpea	/	/	1.50	/	/	0.20	/
	Coffee	/	/	0.02	/	0.01	0.70	0.05
	Bitter melon	/	/	0.50	/	1.00	0.30	0.30
	Pepper	/	0.30	0.50	0.50	2.00	0.40	0.50
	Lychee	/	/	/	/	/	0.01	/
	Potato	/	0.02	0.10	/	0.02	0.02	0.04
	Mango	/	3.00	0.70	/	1.50	3.00	0.70
	Kiwifruit	/	0.70	0.50	/	1.00	/	/
	Loquat	/	0.70	2.00	/	/	3.00	0.70
	Ginseng	/	0.03	/	/	0.10	0.40	/
	Sanchi	/	/	/	/	/	/	/
	Pecan	/	0.02	/	/	/	/	0.04
	Edible rose	/	/	/	/	/	15.00	/
	Rice	/	5.00	3.50	/	/	5.00	2.00
	Peach	/	3.00	0.50	5.00	/	3.00	0.30
	Melon	/	/	0.50	/	1.00	0.30	0.30
	Sunflower	/	/	/	/	/	0.01	/
	Red bayberry	/	/	2.00	/	/	/	/
	Cherry	/	3.00	2.00	/	0.50	/	3.00
	Jujube	/	0.70	2.00	/	3.00	/	/

**Table 4:** The dietary intake risk assessment of tebuconazole and trifloxystrobin.

Food Classification	Fi (kg)	Tebuconazole				Trifloxystrobin			
		Reference Limits (mg/kg)	Sources	NEDI (mg)	RQ (%)	Reference Limits (mg/kg)	Sources	NEDI (mg)	RQ (%)
Rice and its products	0.2399	0.5	China	0.12		0.1	China	0.0240	
Flour and its products	0.1385	0.05	China	0.0069		0.2	China	0.0277	
Other cereals	0.0233	0.05	China	0.0012		0.02	China	0.0005	
Tubers	0.0495	0.1	China	0.005		0.2	China	0.0099	
Darker vegetables	0.0915	15	China	1.3725		0.7	China	0.0641	
Light vegetables	0.1837	7	China	1.2859		0.7	China	0.1286	
Fruits	0.0457	3	China	0.1371		3	China	0.1371	
Nuts	0.0039	0.05	China	0.0002		0.02	CAC	0.0001	
Vegetable oil	0.0327	2	China	0.0654		0.02	China	0.0007	
Salt	0.0120	15	EU	0.18		15	EU	0.1800	
Soy sauce	0.0090	15	China	0.135		0.03	CAC	0.0003	
Total	1.0286			3.3091	175.1			0.5728	22.7

**Table 5:** Environmental parameters of each experimental site in field trials.

Location	GPS	Soil Properties			Climatic		Plant Height (m)	BBCH
		pH	OMC (%)	CEC (cmol/kg)	Mean Temp (°C)	Rainfall (mm)		
Beijing	39.991° N, 116.008° E	7.8	2.3	24.1	24.2	0	1.0–1.5	65
Shanxi	36.734° N, 103.265° E	8.4	1.4	23.9	20.5	5	1.5–2.0	67
Yunnan	25.361° N, 103.495° E	6.9	3.9	11.9	20.7	35	1.2–1.8	66
Shandong	36.251° N, 116.350° E	6.5	2.0	9.3	25.4	2	0.5–1.2	61

OMC: organic matter content; CEC: cation exchange capacity; BBCH: Biologische Bundesanstalt, Bundessortenamt und CHemische Industrie.

## Data Availability

The original contributions presented in this study are included in the article. Further inquiries can be directed to the corresponding author(s).
